# Influence of Bisphenol A on Thyroid Volume and Structure Independent of Iodine in School Children

**DOI:** 10.1371/journal.pone.0141248

**Published:** 2015-10-23

**Authors:** Na Wang, Ying Zhou, Chaowei Fu, Hexing Wang, Peixin Huang, Bin Wang, Meifang Su, Feng Jiang, Hong Fang, Qi Zhao, Yue Chen, Qingwu Jiang

**Affiliations:** 1 Department of Epidemiology, School of Public Health, Key Laboratory of Public Health Safety of Ministry of Education, Fudan University, Shanghai, China; 2 Department of Chronic Disease Control and Prevention, Haimen City Center for Disease Control and Prevention, Haimen, China; 3 Department of Chronic Disease Control and Prevention, Yuhuan County Center of Disease Control and Prevention, Taizhou, China; 4 Department of Chronic Disease Control and Prevention, Shanghai Minhang Center for Disease Control and Prevention, Shanghai, China; 5 School of Epidemiology, Public Health and Preventive Medicine, Faculty of Medicine, University of Ottawa, Ottawa, Canada; Central South University, CHINA

## Abstract

**Background:**

Although several studies have evaluated the relationship between bisphenol A (BPA) and thyroid functions, their results are not entirely consistent. Little is known about BPA in relation to thyroid volume and structure.

**Methods:**

We examined the association of BPA with thyroid volume and thyroid nodules using data from 718 Chinese children living in the East Coast of China in 2012. First morning urine samples were collected for the determination of urinary BPA, creatinine, and urinary iodine concentrations (UIC). Thyroid volume (TV) and nodules were assessed by thyroid ultrasonography.

**Results:**

The median of TV was 3.14ml. 459(63.9%) children took iodized salt at home and the median of UIC was 159μg/l. BPA was detected in 99.9% of the urine samples and the medians for boys and girls were 2.64 and 2.35μg/g creatinine, respectively. Of all participants 14.0% had thyroid nodules. Urinary BPA concentration was inversely associated with thyroid volume (β = -0.033, 95% CI: -0.053, -0.013) and the risk for multiple nodules (OR = 0.78; 95% CI: 0.63, 0.97). The associations above were similar for children who consumed iodized salt and those consumed non-iodized salt.

**Conclusions:**

The data suggest that BPA may be one of the influencing factors for TV and thyroid nodules and its effects are independent of iodine nutrition status in children.

## Introduction

Thyroid volume (TV) is usually measured to evaluate the goiter status and then to assess the degree of iodine deficiency in a population [1]. The influencing factors of TV in children include age, sex, body surface area (BSA), pubertal stage, and iodine nutritional status [2–4]. In addition to iodine nutrition status, other chemicals could also contribute to the development of non-toxic diffuse goiter [5]. Recent epidemiological studies have suggested that Bisphenol A (BPA) disrupts thyroid function in adults [6–9]. BPA is one of the highest production and consumption volume chemicals in the world [8]. Studies from different countries and areas have demonstrated the presence of urinary BPA in more than 90% of their study populations, suggesting a common exposure to BPA worldwide [7, 10–11]. Exposure to BPA is prevalent among children and adolescents [10], who may be more sensitive to its adverse effects including the effect on thyroid function.

The endocrine-disrupting effect of BPA might contribute to numerous complex outcomes including diabetes [12], obesity [13–15], and some other medical disorders [16] such as cognitive and behavioral disorders [17–19]. BPA and other environmental disrupting chemicals (EDCs) may influence the levels of thyroid and steroid hormones and result in increased risks of obesity and some other chronic conditions [20].

Either excess iodine intake or iodine deficiency was associated with an increased risk of developing thyroid nodules [21]. In order to reduce the iodine deficiency, the Universal Salt Iodization (USI) program was launched in China in October 1994 [22]. This program has been implemented for over 20 years and iodized salt remains the main dietary source of iodine. USI has significantly contributed to the prevention of iodine deficiency disorder (IDD) worldwide [23]. However, excess iodine consumption may also have adverse public health impacts [24]. It has been argued that the uniform iodized salt criterion might not have worked across China as expected [22]. The iodine status in children and adults is above an adequate level (100–200μg/L) in some areas and researchers have suggested to reduce the iodine intake accordingly [25]. It is not known if iodine nutrition status has an impact on the association between BPA thyroid measures.

In the current study, we explored the influence of BPA, in addition to iodine, on thyroid volume as well as thyroid nodules. Iodine intake is required for the production of thyroid hormone, and iodine nutrition would change thyroid volume and structure. Both thyroid volume and thyroid nodules [21] can be associated with abnormalities of thyroid function. We therefore also accessed the associations between BPA and thyroid outcomes in children according to their iodine nutrition status.

## Materials and Methods

### Study population

Three primary schools were selected from Minhang District in Shanghai, Haimen City in Jiangsu Province and Taizhou City in Zhejiang Province, respectively. Four classes in each grade from grade 3 to grade 5 in these schools were randomly selected and all students without preexisting thyroid conditions in selected classes were enrolled into this study. Since thyroid examinations were not routinely conducted in children, no student reported preexisting thyroid abnormalities. Among 1267 children enrolled, 1248 provided first morning urine samples and 1234 students completed routine physical examinations. An ultra-sound test for thyroid gland volume was performed on 1064 students. BPA and urinary creatinine were measured for 803 urine samples. After excluding those with no urinary test or thyroid test, we included 718 students in the current analysis. Written consent from parents or guardians of all participants were received and the study was approved by the Ethical Review Board of the School of Public Health of Fudan University.

### Anthropometric measurements

Anthropometric measurements, including standing height (cm), weight (kg), and circumferences of the waist, hip and chest (cm) were taken by trained health professionals according to a standard protocol. The standing height was measured to the nearest 0.1cm without shoes. Weight was measured to the nearest 0.1kg using a digital weight scale. Body mass index (BMI) was calculated as weight in kilograms divided by the square of height in meters and overweight/obese status were assessed using the BMI growth reference values for Chinese children [26].

### Test for thyroid gland volume and thyroid nodules

Students in Taizhou and Haimen received thyroid ultrasonography performed by experienced examiners in local comprehensive hospitals. For students in Minhang, thyroid ultrasonography was performed at school using a real-time sector scanner with a 7.5-MHz/40-mm probe linear transducer. The ultrasonographic examination was carried out on the children lying on a desk with the neck extended. The volume of each lobe was calculated by using the following formula: volume of one lobe (mL) = 0.479*maximum thickness*maximum width*maximum length (cm)[27]. The total thyroid volume was the sum of both lobes and the isthmus volume was not included. Standardized thyroid ultrasound technique was adopted according to the method described by Fuse et al [28]. According to the Chinese national criteria for thyroid measurement, goiter was defined by age-specific thyroid volume. The upper limits of thyroid volume for children aged 9, 10, and 11 years were 5.0, 6.0 and 7.0, respectively. If thyroid volume exceeded the relevant limit, a child was judged as being goitrous [29]. Discrete lesion(s) within the thyroid gland that is palpably and / or ultrasonographically distinct from the surrounding thyroid parenchyma were defined as thyroid nodule(s) [30]. It was also detected by ultrasonography in our study. In case of abnormality in the sonographic examination of the thyroid, parents of the children would receive a written note describing the abnormal results of the examination.

### Urine and salt samples collection and iodine concentration analyses

The day prior to a physical examination, a 50ml BPA free tube was distributed to each student. First morning urine samples were collected in children's home and brought to school by students themselves in the next morning. The collected urine samples were kept frozen at -80°C until analysis. Students were also asked to bring a salt sample of more than 20g from home for iodine measurement.

Urinary iodine concentrations (UICs) were determined following the method proposed by the Ministry of Health of the People's Republic of China (WS/T107. 2006, and GB/T13025.7–1999) [27]. Salt iodine content was also measured using a national standard method with a proper quality control [31](GB/T 13025.7–1999). 10% urine samples were assayed in duplicate.

### Analyses of urine BPA

Details of the assays for the detection of BPA have been published previously [32]. Briefly, after an aliquot (1.0 mL) of urine sample was enzymatically hydrolyzed, BPA was purified and separated by reversed-phase and anion-exchange mixed-mode solid-phase extraction. It was analyzed by ultra-performance liquid chromatography coupled with tandem mass spectrometry. It was derivatized by dansyl chloride, and then resolved by the mobile phase of methanol and water both containing 10 mM of ammonium formate on reversed-phase ultra-performance liquid chromatographic C18 column and ionized in positive ion mode. BPA was quantified by using an isotope internal standard curve method.

All the analyses of urine samples were completed in twelve batches within two months. Four spiked urine samples with BPA at the concentration of 5 ng/mL and four solvent blanks were prepared along with each batch to monitor the background values of phthalate metabolites and the accuracy of analytical procedure. The inter-batch recoveries of BPA of spiked urine samples varied between 81% and 121% with the inter-batch relative standard deviations (RSDs) ranging from 6% to 16%.

Urinary creatinine concentration in each sample was also measured using an enzymatic method. BPA concentrations were then adjusted for urinary dilution by creatinine levels.

### Statistical analysis

Values below the method limit of detection (LOD) for BPA (0.06ng/ml) were replaced with a value of the LOD divided by the square root of 2 [33]. Since data were not normally distributed, their logarithmically transformed values were therefore used, including thyroid volume, BPA, BSA(BSA = 0.007184*weight^0.425^*height^0.725^) and BMI. One-way analysis of variance (ANOVA) was performed to test the difference in medians of thyroid volume and urinary BPA concentration between groups categorized by sex, age, BSA (in tertiles), iodized salt consumption status, urinary iodine level and study area. Simple linear regression analysis was used to examine the correlations between thyroid volume and its determinants. Multivariable linear regression models were used to adjust for age, sex, iodized salt consumption, BSA (log-transformed) and BMI (log-transformed). The median levels of log-transformed BPA across quintiles of TV were calculated and then compared using ANOVA. Due to a considerable day-to-day variation in iodine excretion, one-spot urinary iodine level was not a proper indicator of iodine status for individuals [34]. Therefore, in current analysis, iodized salt consumption instead of iodine concentration in urine, was included in the multivariate models. We also assessed the correlation between UIC and iodized salt consumption and observed a significantly higher level of UIC in children who consumed iodized salt at home, suggesting that iodized salt consumption status could be a good proxy for iodine nutrition at a population level.

The association of urinary BPA concentration (in quintiles) with thyroid nodules (no, solitary nodule, and multiple nodules) was assessed using multinomial logistic regression analysis. All analyses were performed by using SAS, version 9.3 software (SAS Institute, Inc., Cary, NC,USA), and all tests of statistical significance were base on two-side probability.

## Results

### Participant characteristics

A total of 1267 students were recruited for this survey and 718 of them were included in the present analysis. The demographic characteristics as well as iodine nutrition status were summarized in Table 1. There were no statistically significant differences in sex and iodized nutrition status between children included in this analysis (N = 718) and those excluded (549). The median of UIC for children included in the analysis was 159μg/l.

**Table 1:** Characteristics of participants in the analysis and non-participants.

Characteristics	Non-participants		Participants in the analysis		χ^2^	P-value
	N	%	N	%		
Total	549	100.00	718	100.00		
Sex					1.431	0.232
Male	262	47.72	367	51.11		
Female	287	52.28	351	48.89		
Age					75.772	<0.0001
9	119	21.68	321	44.71		
10	234	42.62	228	31.75		
11	196	35.70	169	23.54		
BMI ^a^					7.063	0.029
Normal	379	69.03	560	77.99		
Overweight	89	16.21	93	12.95		
Obese	56	10.20	57	7.94		
Iodized salt consumption ^b^					0.807	0.369
No	172	31.33	259	36.07		
Yes	340	61.93	459	63.93		
Urinary iodine(μg/l) ^c^					0.935	0.626
<100	131	23.86	167	23.26		
100–200	225	40.98	297	41.36		
>200	174	31.69	254	35.38		

^a^: 25 missing

^b^: 37 missing

^c^: 19 missing

### Thyroid volume measurements

The median thyroid volume of participants was 3.14ml, and was similar for boys (3.05ml) and girls (3.21ml). Thyroid volume significantly increased with age, BSA and urine iodine concentration (Table 2). Children consuming iodized salt had a relatively larger thyroid gland volume as compared with those consuming non-iodized salt. The proportions of children consuming iodized salt in Minhang, Haimen and Taizhou were 75%, 94% and 25%, respectively, which partly explained a significantly lower level of TV in Taizhou. According to the thyroid volume-based criteria by age for screening of goiter in children, 6% (15 boys and 25 girls) had goiter.

**Table 2:** Thyroid volume and urinary BPA concentration of participants characterized by sex, age, BSA, area and iodine nutrition status.

Characteristics	Thyroid Volume		Urinary BPA concentration ^a^	
	Median (IQR)	*P*-value	Median (IQR)	*P*-value
All	3.14(2.44–4.11)		2.45(1.09–5.97)	
Sex		0.263		0.126
Male	3.05(2.43–4.03)		2.64(1.13–6.40)	
Female	3.21(2.45–4.25)		2.35(1.04–5.40)	
Age (years)		<0.0001		0.028
9	2.92(2.25–3.85)		2.24(1.04–5.34)	
10	3.74(2.75–4.70)		2.54(0.98–6.41)	
11	3.02(2.46–3.58)		2.89(1.27–6.34)	
BSA^b^		<0.0001		0.050
T1 (<1.06 m^2^)	2.57(2.05–3.45)		2.26(1.01–5.12)	
T2 (1.06–1.19 m^2^)	3.31(2.59–4.41)		2.49(1.05–5.58)	
T3 (> = 1.20 m^2^)	3.42(2.70–4.54)		3.02(1.27–6.85)	
Iodized salt consumption		<0.0001		<0.0001
No	2.73(2.16–3.50)		3.44(1.74–7.02)	
Yes	3.35(2.61–4.43)		2.14(0.85–5.21)	
Urinary iodine(μg/l)		0.0003		0.783
<100	2.75(2.21–3.55)		2.29(1.10–4.58)	
100–200	3.18(2.45–4.11)		2.44(1.02–6.35)	
>200	3.33(2.55–4.43)		2.49(1.15–6.36)	
Area		<0.0001		<0.0001
Minhang	4.25(3.44–5.17)		1.74(0.94–3.43)	
Haimen	3.13(2.46–4.07)		2.22(0.78–5.20)	
Taizhou	2.52(2.02–3.07)		3.95(2.04–10.48)	

BPA: Bisphenol A; BSA: Body Surface Area; IDR: Inter-quartile range

^a^: Creatinine adjusted (μg/mg)

^b^: In tertiles

### Urinary BPA concentrations

BPA was detected in 99.9% of the urine samples. Median BPA concentrations were similar for boys (2.64μg/g creatinine) and girls (2.35μg/g creatinine), but increased with age (p-trend = 0.028) (Table 2). The urinary BPA concentration also showed a geographic difference. The median BPA concentration was the lowest in Minhang (1.74μg/g creatinine), the highest in Taizhou (3.95μg/g creatinine), and in between in Haimen (2.22μg/g creatinine) (Table 2).

### Association between urinary BPA concentration and thyroid volume

**Fig 1:**
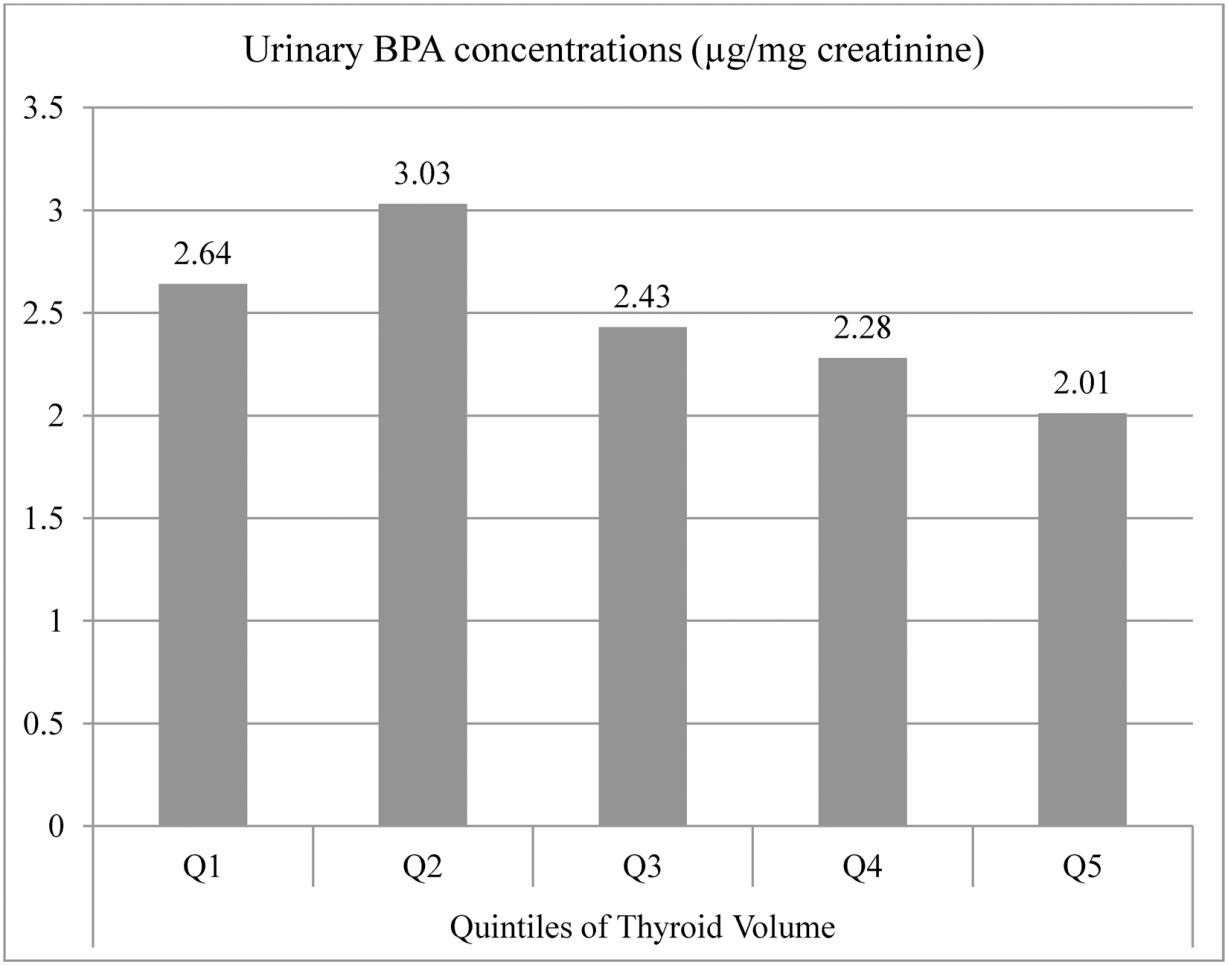
Median (μg/mg creatinine) value of urine BPA concentrations after urine creatinine correction, by quintiles of Thyroid volume (ml).

There was a significant inverse association between BPA concentration and TV (Fig 1). A simple linear regression model for the association showed a regression coefficient (β) of -0.036 (95% CI: -0.056, -0.016). After adjustment for age, sex, (ln)BSA and iodized salt consumption status, the association persisted (Table 3). Each log unit increase in BPA level was associated with a 0.088 SD unit decrement in thyroid volume. When we used BMI instead of BSA, and used urinary iodine concentration instead of iodized salt consumption as covariates, the inverse association between thyroid volume and BPA remained significant. In addition, the inverse associations did not differ significantly by sex, or iodized salt consumption status (Table 3). Urinary concentration of BPA was not significantly associated with the risk of goiter while iodized salt consumption was related to a reduced risk for goiter (Odds Ratio (OR): 0.34; 95% CI: 0.14–0.84) (data not shown).

**Table 3:** Linear regression analysis for association of thyroid volume with BPA (both log-transformed).

		Model	β	95%CI		*P*-value
All participants	Univariate analysis	Model 1^a^	-0.036	-0.056	-0.016	<0.0001
	Multivariate analysis	Model 2^b^	-0.038	-0.058	-0.018	<0.0001
		Model 3^c^	-0.033	-0.053	-0.013	0.001
		Model 4^d^	-0.037	-0.056	-0.018	<0.0001
Sex	Male	Model 3^c^	-0.039	-0.064	-0.015	0.002
	Female	Model 3^c^	-0.032	-0.062	-0.003	0.034
Iodized salt consumption	Yes	Model 3^c^	-0.029	-0.062	0.003	0.074
	No	Model 3^c^	-0.037	-0.061	-0.014	0.002

^a^: Univariate regression analysis

^b^: Adjusted for sex and age only

^c^: Adjusted for sex, age, (ln)BSA, and iodized salt consumption status

^d^: Adjusted for sex, age, (ln)BMI, and urinary iodine concentration(categorical variable: < 100μg/l, 100–200μg/l and >200μg/l)

### Prevalence of thyroid nodules

Thyroid nodules were detected in 100 children, accounting for 14% of all participants. Most nodules were accompanied by hypoechogenicity. The prevalence of multiple thyroid nodules was 6.9%, compared with 7.1% for solitary nodules. The risk for thyroid nodules was negatively associated with age but showed no sex related difference. There was no association of thyroid nodules with urinary iodine level or iodized salt consumption status (Table 4).

**Table 4:** Prevalence of thyroid nodules in participants categorized by sex, age, and iodine nutrition.

Characteristics	N		Thyroid nodules		*P*-value
		No nodule (N (%))	Solitary nodule (N (%))	Multiple nodules (N (%))	
All	718				
Sex					0.413
Male	367	321(87.47%)	21 (5.72%)	25 (6.81%)	
Female	351	297(84.61%)	28 (7.98%)	26 (7.41%)	
Age					0.004
9	321	287(89.41%)	21 (6.54%)	13 (4.05%)	
10	228	191(83.78%)	19 (8.33%)	18 (7.89%)	
11	169	140(82.84%)	9 (5.33%)	20 (11.83%)	
Iodized salt consumption					0.510
No	259	228(88.03%)	16 (6.18%)	15 (5.79%)	
Yes	459	390(84.97%)	33 (7.19%)	36 (7.84%)	
Urinary iodine(μg/l)					0.202
<100	167	144(86.22%)	11 (6.59%)	12 (7.19%)	
100–200	297	247(83.17%)	23 (7.74%)	27 (9.09%)	
>200	254	228(89.77%)	14 (5.51%)	12 (4.72%)	
Area					<0.0001
Shanghai	200	150(75.00%)	25 (12.50%)	25 (12.50%)	
Haimen	261	230(88.12%)	13 (4.98%)	18 (6.90%)	
Taizhou	257	238(92.61%)	11 (4.28)	8 (3.11%)	

### Associations between urinary BPA concentrations and thyroid nodules

Urinary BPA concentration was negatively associated with the risk of multiple nodules, but not with the risk of solitary nodules before and after controlling for sex, age, iodized salt consumption and BMI (in quintiles). There was a reduced risk for multiple nodules with increasing BPA, with an OR being 0.78 (95% CI: 0.63, 0.97). No notable variations in OR estimates were observed across sex, age, and iodized salt consumption categories (Table 5).

**Table 5:** Associations of urinary BPA concentration with thyroid nodules.

		Solitary nodule	Multiple nodules
		OR (95%CI) ^a^	OR (95%CI) ^a^
All subjects	Univariate analysis	0.98 (0.79, 1.20)	0.77 (0.63, 0.95)
	Multivariate analysis ^b^	0.98 (0.79, 1.20)	0.76 (0.61, 0.94)
Sex ^b^	Male	1.07 (0.79, 1.47)	0.82 (0.61, 1.09)
	Female	0.90 (0.68, 1.21)	0.69 (0.49, 0.96)
Age(years) ^b^	9	1.20 (0.87, 1.66)	0.68 (0.43, 1.09)
	10	0.90 (0.64, 1.26)	0.74 (0.52, 1.06)
	11	0.72 (0.43, 1.22)	0.80 (0.56, 1.15)
Iodized salt consumption ^b^	Yes	1.01 (0.79, 1.30)	0.74 (0.57, 0.96)
	No	0.92 (0.62, 1.35)	0.79 (0.53, 1.19)

^a^: Compared with participants with no thyroid nodules

^b^: The results of multiple analysis adjusting for age, sex, BSA (in quintiles), and iodized salt consumption state

## Discussion

In this study of school children conducted in east coast China, we observed diversity in thyroid volume with varied iodine nutrition status. Urinary BPA concentration was inversely associated with thyroid volume, but not with goiter. An increased BPA level was also associated with a reduced risk for multiple thyroid nodules.

Studies that measured thousands of individuals from several different countries overwhelmingly detected BPA in adults, adolescents, and children [35]. Considering the endocrine disruptor effect of BPA and its common exposure, many studies have focused on the potential effects of BPA on various chronic conditions, including diabetes, obesity [14–15], as well as thyroid functions [7].

Epidemiologic studies have revealed an association between BPA exposure and altered thyroid hormones. A cross-sectional study conducted in Shanghai examined this association in 3394 subjects and found increased free triiodothyronine (FT3) and decreased TSH with increased urinary BPA [7]. High urinary BPA level was associated with increased thyroid function (adjusted OR: 1.71, 95% CI: 1.26, 2.32)[7]. The NHANES study also reported a suggestive inverse relationship between urinary BPA and total T4 and TSH among adults [6]. Another survey in a Thai population observed a significantly negative correlation between serum BPA and FT4 level in males only, but BPA was not associated with TSH in either males or females [8]. The CHAMACOS study revealed that exposure to BPA during pregnancy was related to reduced T4 in pregnant women and decreased TSH in male neonates [36]. BPA was observed to have a direct effect on thyroid follicular cell and leads to an altered expression of the genes involved in thyroid hormones synthesis both in vitro and in vivo (zebrafish) models [37]. Other potential mechanisms for the association between BPA and thyroid hormones include inhibiting T3 pathways during metamorphosis [38] and thyroid hormone receptor (TR) transcription suppression [39].

Children are commonly exposed to BPA [10, 19], and those having higher BPA levels experience an elevated risk for being obese/overweight [14, 40]. Obesity is now becoming a great public health concern and is closely linked to numerous adverse health outcomes. Studies suggest that altered thyroid function may be one of the reasons for the relationship between BPA and obesity [41]. However, the association of BPA with abnormality in thyroid morphology and structure has not been well studied previously.

The main determinants of thyroid volume in both boys and girls have been shown to be age, urinary iodine, BSA, and pubertal stage. The findings of our study of Chinese children that thyroid volume was related with age, BSA, and iodine nutritional status are in a general agreement with those of previous studies conducted among other populations [2]. In another study conducted in Zhejiang Province, China, age, sex and BSA were the influencing factors for thyroid volume in children aged 6–12 years, whereas urinary iodine concentration had little effect on the TV [3]. In the study conducted in healthy Greek children by Kaloumenou et al [2], main determinants of thyroid volume in children were age, body surface area, and pubertal stage. The prevalence of thyroid nodule was also significantly increased with age and showed a female predominance [42]. Another study conducted in China indicated that excess iodine intake or iodine deficiency was also risk factors for thyroid nodules [21].

Decreased secreting of thyroxin and subsequent rise in TSH may explain the association of goiter and iodine deficiency [25]. BPA suppresses TSH release from pituitary in a manner independent of both the thyroid hormone feedback mechanism and the estrogenic activity of BPA [6]. Therefore, the inverse association between thyroid volume and BPA observed in our study may partly due to the negative correlation between BPA and TSH. Children exposed to a higher level of BPA may have a reduced TSH and then prohibited the enlargement of thyroid gland. The exposure of BPA may also alter thyroid functions, leading to thyroid abnormalities, such as subclinical hypothyroidism. The risk for developing thyroid nodules could therefore be changed [21], which might partly explain the association of BPA with thyroid nodules.

The sex difference in the BPA effect on the prevalence of thyroid nodules was consistent with findings from experimental studies and previous epidemiological studies on its effects on other chronic outcomes. BPA was associated with overweight/obesity among girls aged 9–12, but the association was not observed among boys [14]. A prospective birth cohort documented that BPA was detected in more than 97% of the gestational and childhood urine samples. The magnitude of the gestational BPA associations differed according to child’s sex, gestational BPA exposure affected behavioral and emotional regulation domains in children at 3 years of age, especially among girls [19]. Wang et al conducted a cross-sectional study in children aged 8–15 and found a significant association between urinary BPA and BMI in females but not in males. [40] This sex-related discrepancy is possibly related to androgen-related differences in the metabolism of BPA [8]. It is also argued that BPA is estrogenic and capable of disrupting sex differentiation [38].

The effects of BPA on TV or thyroid volume were similar in participants consuming iodized salt and those consuming non-iodized salt, which suggested that the effects of BPA on TV and thyroid nodules were independent of iodine exposure.

The association of thyroid volume or thyroid echostructure with other chemicals in addition to iodine nutrition status, has been seldom explored [43]. To our knowledge, this is the first study on the potential effect of BPA on thyroid volume and thyroid nodules. Our study has some limitations. As a cross-sectional study, we did not have repeated measures of the urinary BPA concentration and thyroid volume overtime and did not evaluate long-term effects of BPA on TV. Information on the amount of salt consumed at home and outside of home was not collected and we can only use iodized salt consumption status as proxy of iodine nutrition, which was another limitation of our study. In addition, we had no information on thyroid hormones and were unable to evaluate whether these factors might explain the observed associations.

In conclusion, we observed an inverse association between urinary BPA with thyroid volume and risk for multiple thyroid nodules in children living in the East Coast of China. Further studies are needed to investigate the temporal relationship and potential public health implications of such an association.
